# Ten-year atherosclerotic cardiovascular disease risk prediction for postmenopausal women: Impacts of isolated postchallenge hyperglycemia

**DOI:** 10.1097/MD.0000000000030352

**Published:** 2022-09-09

**Authors:** Tsung-Hui Wu, Yi-Chun Lin, Chii-Min Hwu

**Affiliations:** a Section of Endocrinology and Metabolism, Department of Medicine, Taipei Veterans General Hospital, Taipei, Taiwan; b Faculty of Medicine, National Yang Ming Chiao Tung University School of Medicine, Taipei, Taiwan; c Rong Yang Clinic, Taipei, Taiwan.

**Keywords:** atherosclerosis, cardiovascular disease, hyperglycemia, menopause, risk assessment

## Abstract

Isolated postchallenge hyperglycemia (IPH) is a type of diabetes mellitus defined as 2-h glucose ≥200 mg/dL but fasting glucose <126 mg/dL. The purpose of the study was to assess impacts of IPH on 10-year atherosclerotic cardiovascular disease (ASCVD) risk scores in postmenopausal women. This study analyzed data from 428 postmenopausal women who underwent oral glucose tolerance test at a medical center. Ten-year ASCVD risk was evaluated by using Pooled Cohort Equations. Logistic regression analysis was performed to estimate odds ratios for having high 10-year ASCVD risk scores (≥5%) among these women. The subjects with IPH had higher systolic blood pressure and worse lipid profile than those without IPH. Ten-year ASCVD risk scores for postmenopausal women with IPH were calculated under 2 scenarios: the IPH women were considered non-diabetic, they were designated as patients with DM. The median ASCVD risk score of the participants with IPH increased significantly from 3.7% under scenario 1 to 7.1% under scenario 2. Approximately 20% women with IPH were re-categorized from risk category of <5% to ≥7.5% once they were identified as patients with DM (scenario 2). The results of logistic regression analyses showed that IPH was independently positively associated with 10-year ASCVD risk scores ≥5% under both scenarios. Postmenopausal women with IPH were characterized by unfavorable cardiovascular risk profile and high predicted 10-year ASCVD risk. Knowing the women’s hidden DM status would significantly alter their risk categorization.

## 1. Introduction

Using risk calculators to estimate atherosclerotic cardiovascular disease (ASCVD) risk is a well-accepted method of risk stratification for apparently healthy, asymptomatic individuals.^[1]^ Recently, the American College of Cardiology (ACC) and American Heart Association (AHA) published an updated guideline for the assessment of cardiovascular risk for primary prevention of ASCVD.^[2]^ This state-of-art guideline provided a new risk calculator, the Pooled Cohort Equations,^[3]^ to predict 10-year risk of “hard” ASCVD events (including nonfatal myocaridal infarction, fatal coronary heart disease, and fatal and nonfatal stroke) in adults without known cardiovascular disease. The variables that included in this risk assessment tool are age, race, total cholesterol, high-density lipoprotein (HDL) cholesterol, status of diabetes mellitus (DM), current smoking status, systolic blood pressure (BP), and whether the individual is treated for hypertension.^[3]^

While the novel ACC/AHA assessment instrument expands the endpoint for ASCVD risk prediction and widens the range of applicable ethnic backgrounds,^[4]^ it introduces uncertainties in ASCVD risk assessment as well.^[5]^ For example, the variable of DM status is dichotomous (yes or no) in the equations, but individuals with DM detected only by oral glucose tolerance test could not be readily identified in clinical practice. The uncertainty in DM status may alter the results of risk stratification.

Patients can be diagnosed with DM by fasting plasma glucose levels ≥126 mg/dL, but fasting plasma glucose levels below this threshold do not necessarily exclude the presence of DM.^[6]^ Oral glucose tolerance test is 1 of the recommended screening tests for DM, and those with 2-hour plasma glucose levels ≥200 mg/dL during the test will be diagnosed with DM.^[6]^ Isolated postchallenge hyperglycemia (IPH) is a type of DM with fasting plasma glucose <126 mg/dL and 2 h postchallenge glucose ≥200 mg/dL after 75-g oral glucose loading.^[7]^ Yang et al^[8]^ found that more than 50% of the women with undiagnosed DM in China, mostly middle-aged, had IPH. Barrett-Connor and Ferrara emphasized that older women with IPH were more vulnerable to fatal cardiovascular disease than men with IPH.^[9]^ These burdens have made IPH a significant health issue for postmenopausal women. However, although IPH has important consequences for women’s cardiovascular health, it is generally unrecognized by practitioners because the diagnosis of IPH is relied on oral glucose tolerance test.^[10]^ Unawareness of the hidden DM status would be a pitfall in the implementation of the Pooled Cohort Equations for these women.

In the present study, we determined 10-year ASCVD risk scores in a group of naturally postmenopausal women. Frequency distribution of the 10-year ASCVD risk score categories was investigated among the subjects. To survey the influences of awareness of IPH on ASCVD risk estimation, we computed the IPH women’s 10-year risk scores under 2 scenarios: the IPH women were considered non-diabetic, they were designated as patients with DM. The levels of predicted ASCVD risk between the 2 scenarios were compared in the study. In addition, we were also interested in exploring the impacts of IPH on atherosclerotic cardiovascular risk stratification for these women.

## 2. Materials and Methods

### 2.1. Subjects

This study investigated data from the Veterans Hospital Study of Oral Glucose Tolerance (the VSO), a clinical data registry focusing on information of subjects who underwent oral glucose tolerance tests at the Taipei Veterans General Hospital. Details of the VSO have been reported elsewhere.^[11]^ The Institutional Review Board of the Taipei Veterans General Hospital approved each individual protocol and each participant gave written informed consent before entering the study.

For the purpose of this study, we analyzed data from 428 naturally postmenopausal women who had not menstruated within the last 12 months and aged between 40 and 75 years old. Women with a known history of DM or fasting glucose higher than 126 mg/dL were excluded from the study. The subjects could not have a history of major systemic disease except hypertension. None of them had acute illness in the past 6 months. None of the participants reported taking concomitant glucose-modification agents and anti-lipid drugs.

### 2.2. Measures

The participants were asked to visit our clinics at 8 AM after an 8 to 10 hour overnight fast. All subjects received a detailed history taking and a brief physical examination after measurements of weight, height, and BP. Fasting blood samples were collected for the measurements of plasma glucose, lipids, and biochemistry. Then, the subjects were asked to drink 300 mL of a sugary solution that contains 75 g of glucose monohydrate in 5 minutes. Blood samples were taken at 120 minutes after glucose loading to examine if they had IPH. Plasma glucose was measured by a glucose oxidase method in a glucose analyzer (model 2300; YSI, Yellow Springs, OH). Serum lipids and biochemistry were measured by using commercial assay kits in an automatic blood chemistry analyzer.

### 2.3. Calculation

Body mass index (BMI) was calculated as weight divided by height squared (kg/m^2^). A physical inactivity score was calculated using the formula reported previously: (hours of sedentary activity)/(24 hours—hours of basal activity).^[12]^ We calculated the 10-year ASCVD risk score for each woman based on age, systolic BP, treatment of hypertension, total and HDL cholesterol levels, current smoking, and status of DM, using the sex-specific parameters from the ACC/AHA Pooled Cohort Equations.^[3]^ The subjects with normal glucose tolerance (NGT) and impaired glucose tolerance (IGT) were defined as non-diabetic for calculating 10-year ASCVD risk. Ten-year ASCVD risk scores of the women with IPH were calculated under 2 scenarios: the IPH women were assigned as non-diabetic; they were designated as patients of DM. In addition, we compared the differences between the 2 scenarios in categorizing the IPH women according to 10-year risk thresholds of 5% and 7.5%.^[2]^

### 2.4. Statistical analysis

Data are expressed as mean (SD), median (range), or n (%). The participants were divided into 3 groups by status of oral glucose tolerance: NGT, IGT, and IPH. Because of their skewed distribution, fasting triglycerides (TG), alanine aminotransferase (ALT), and 10-year ASCVD risk score were analyzed after logarithmic transformation. Clinical and biochemical characteristics were compared using *χ*^2^ tests and 1-way analysis of variance with a post hoc Duncan test when the variables were categorical and continuous, respectively. Odds ratios and 95% confidence intervals of increased 10-year risk scores (≥5% vs <5%) among women with different oral glucose tolerance status were estimated from univariate logistic regression models using the NGT group as the reference (odds ratio = 1). The relative contributions of BMI and other covariates to the association of high ASCVD risk score (≥5%) with IPH were evaluated by multinomial logistic regression. The following covariates were entered compulsorily into the multinomial model: BMI, physical inactivity score, Log ALT, serum Cr, treatment of hypertension (yes vs no), and hormone replacement therapy (yes vs no). Statistical analysis was performed using Statistical Package for Social Sciences software (Version 18.0; SPSS Inc., Chicago, IL) and STATA program (Version 12.1; StataCorp, College Station, TX). A *P* value < .05 was considered statistically significant.

## 3. Results

The clinical characteristics of the study subjects by glucose tolerance status are shown in Table 1. The present study included 78 subjects with IPH, 214 with IGT, and 136 with NGT. The 3 groups were comparable in values of total cholesterol, low-density lipoprotein (LDL) cholesterol, serum creatinine, and physical inactivity score. Women with glucose intolerance (IGT and IPH) were older than women with NGT. Women with IPH had higher values of BMI, systolic BP, Log ALT, Log TG, and fasting glucose and lower levels of HDL cholesterol than those without IPH (Table 1).

**Table 1:** Descriptive characteristics of the study participants by glucose tolerance status.

	NGT	IGT	IPH	*P*
n	136	214	78	–
Age (yr)	56.9 (5.0)	58.3 (5.0)*	59.6 (5.5)*	.001
BMI (kg/m^2^)	23.4 (3.0)	24.0 (3.4)	24.9 (4.0)*,†	.008
Physical inactivity scores	0.69 (0.21)	0.69 (0.19)	0.74 (0.19)	.15
Hypertensive, n (%)	20 (14.7)	45 (21.0)	22 (28.2)	.058
Hormone replacement therapy, n (%)	9 (6.6)	12 (5.6)	1 (1.3)	.21
Current smoking, n (%)	2 (1.5)	2 (0.9)	2 (2.6)	.58
Systolic BP (mm Hg)	116 (19)	122 (19)*	129 (20)*,†	<.0001
Diastolic BP (mm Hg)	69 (10)	71 (10)*	73 (11)*	.008
ALT (U/L)	18 (63)	20 (165)	23 (185)	–
Log ALT	1.27 (0.15)	1.33 (0.20)*	1.40 (0.21)*,†	<.0001
Cr (mg/dL)	0.64 (0.15)	0.63 (0.16)	0.63 (0.14)	.66
Total cholesterol (mg/dL)	210 (34)	209 (34)	214 (31)	.50
TG (mg/dL)	82 (331)	98 (379)	121 (429)	–
Log TG	1.93 (0.20)	2.00 (0.20)*	2.08 (0.20)*,†	<.0001
HDL cholesterol (mg/dL)	65 (16)	60 (15)*	56 (13)*,†	<.0001
LDL cholesterol (mg/dL)	131 (32)	133 (34)	138 (31)	.33
Fasting glucose (mg/dL)	93 (7)	98 (8)*	103 (9)*,†	<.0001
2 h glucose (mg/dL)	124 (12)	164 (16)*	229 (23)*,†	<.0001

Data are expressed as mean (SD), median (range), or n (%). The medians (ranges) of ALT and TG values are also presented in the table. One-way analysis of variance or *χ*^2^ tests were used to compare the differences among the 3 groups.

ALT = alanine aminotransferase, BMI = body mass index, BP = blood pressure, Cr = creatinine, HDL = high-density lipoprotein, IGT = impaired glucose tolerance, IPH = isolated postchallenge hyperglycemia, LDL = low-density lipoprotein, Log ALT = logarithmic transformation of ALT (in U/L), Log TG = log transformation of fasting TG (in mg/dL), NGT = normal glucose tolerance, TG = fasting triglycerides.

* *P* < .05 versus NGT; compared by Bonferroni correction method.

† *P* < .05 versus IGT; compared by Bonferroni correction method.

**Figure 1.**
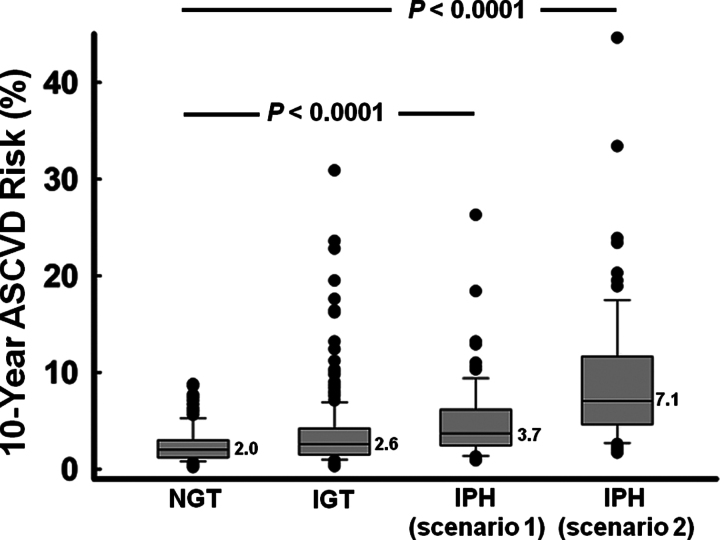
Box plots of 10-year ASCVD scores in postmenopausal women with different glucose tolerance status. Ten-year ASCVD risk scores differed significantly among the 3 groups with higher values in the group with IPH, no matter which scenario was applied to the women with IPH (both *P* < .0001). The number next to the line in each box plot indicates the median. The boundary of the box closest to zero indicates the 25th percentile and the boundary of the box farthest from zero indicates the 75th percentile. Whiskers (error bars) above and below the box indicate the 90th and 10th percentiles. Filled circles (●) indicate outlying points (a value more than 90th percentile or <10th percentile). The *P* value indicates the statistical significance of ASCVD scores among the women with different glucose tolerance status. Scenario 1: if IPH undiagnosed; Scenario 2: if IPH diagnosed. ASCVD = atherosclerotic cardiovascular disease, IGT = impaired glucose tolerance, IPH = isolated postchallenge hyperglycemia, NGT = normal glucose tolerance.

The distribution of predicted 10-year ASCVD risk (%) is presented in Figure 1, using box plots and stratified by oral glucose tolerance status. The number next to the line in each box plot indicates the median. Ten-year ASCVD risk scores differed significantly among the 3 groups with higher values in the IPH group, no matter which scenario was applied to the women with IPH (both *P* < .0001).

**Figure 2.**
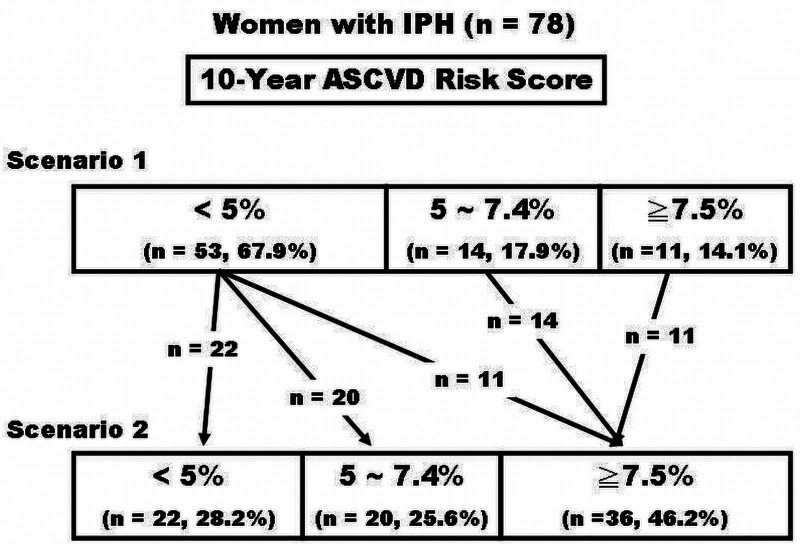
A substantial number of women with isolated postchallenge hyperglycemia (IPH) were reclassified into a higher risk group when they were labeled as diabetes mellitus in calculating the 10-year atherosclerotic cardiovascular disease (ASCVD) scores. Scenario 1: if IPH undiagnosed; Scenario 2: if IPH diagnosed.

Under scenario 1, 14% (n = 11) women in the IPH group had 10-year risk scores more than 7.5% and another 18% (n = 14) had the scores between 5% and 7.5% (Table 2). Under scenario 2, the proportion of subjects with 10-year ASCVD risk ≥7.5% in the IPH group increased 3-fold to 46% (n = 36), and the proportion with risk scores between 5% and 7.5% increased 1.4-fold to 25.6% (n = 20) (Table 2). We also noted that knowing the women’s hidden DM status (that is, under scenario 2) would significantly alter their risk categorization. A substantial number of women with IPH (45 out of 78, 57.6%) were reclassified into a higher risk group when they were labeled as DM in calculating the ASCVD risk scores (Fig. 2, scenario 2). Eleven out of 53 IPH women (20.8%) with 10-year risk score <5% initially (scenario 1) were re-categorized to risk category of ≥7.5% once the women were identified as patients with DM (scenario 2) (Fig. 2).

**Table 2:** Frequency distribution of the 10-year risk score categories for atherosclerotic cardiovascular disease among postmenopausal women with different oral glucose tolerance status.

	NGT	IGT	IPH*	IPH†
10-year ASCVD risk score				
<5%	121 (89.0)	171 (79.9)	53 (67.9)	22 (28.2)
5%–7.4%	11 (8.1)	23 (10.7)	14 (17.9)	20 (25.6)
≥7.5%	4 (2.9)	20 (9.3)	11 (14.1)	36 (46.2)

Data are expressed as subject number (%).

ASCVD = atherosclerotic cardiovascular disease, IGT = impaired glucose tolerance, IPH = isolated postchallenge hyperglycemia, NGT = normal glucose tolerance.

* Subjects with IPH were not designated as patients of diabetes mellitus while performing ASCVD risk estimation (scenario 1).

† Subjects with IPH were designated as patients of diabetes mellitus while performing ASCVD risk estimation (scenario 2).

The relationship between increased 10-year risk score (≥5%) and IPH was explored by logistic regression analysis (Table 3). Under scenario 1, the women with IPH had more than tripled the risk of high 10-year risk scores (≥5%) (odds ratio 3.81; 95% CI 1.86–7.79, *P* < .0001) compared to those with NGT. The positive association between IPH and high 10-year risk scores was maintained despite BMI and other covariates were added to the regression model for adjustments. Under scenario 2, the odds ratio of having high 10-year risk scores (≥5%) for women with IPH was 20.53 (95% CI 9.91–42.55, *P* < .0001) before adjustment and 26.74 (95% CI 11.47–62.34, *P* < .0001) after adjustments for multiple covariates, respectively.

**Table 3:** Crude and adjusted odds ratios (95% CI) of increased 10-year risk scores (≥5%) for atherosclerotic cardiovascular disease in postmenopausal women according to oral glucose tolerance status.

	NGT	IGT	IPH
Scenario 1†			
No. of subjects (10-yr ASCVD risk score ≥5% vs <5%)	15/121	43/171	25/53
Model 1	1.0	2.03 (1.08–3.82)*	3.81 (1.86–7.79)***
Model 2	1.0	1.92 (1.01–3.62)*	3.35 (1.62–6.92)**
Model 3	1.0	1.96 (0.98–3.89)	3.28 (1.47–7.36)**
Scenario 2‡			
No. of subjects (10-yr ASCVD risk score ≥5% vs <5%)	15/121	43/171	56/22
Model 1	1.0	2.03 (1.08–3.82)*	20.53 (9.91–42.55)***
Model 2	1.0	1.89 (0.99–3.58)	19.03 (9.08–39.90)***
Model 3	1.0	1.87 (0.93–3.78)	26.74 (11.47–62.34)***

Data are expressed odds ratios (95% CI).

Model 1, no adjustment; Model 2, further adjusted for BMI; Model 3, further adjusted for physical inactivity score, Log ALT, serum Cr, hypertension treatment (yes vs no), and Hormone replacement (yes vs no).

ALT = alanine aminotransferase, ASCVD = atherosclerotic cardiovascular disease, BMI = body mass index, CI = confidence intervals, Cr = creatinine, IGT = impaired glucose tolerance, IPH = isolated postchallenge hyperglycemia, Log ALT = logarithmic transformation of ALT (in U/L), NGT = normal glucose tolerance.

† Scenario 1: subjects with IPH were not designated as patients of diabetes mellitus while performing ASCVD risk estimation.

‡ Scenario 2: subjects with IPH were designated as patients of diabetes mellitus while performing ASCVD risk estimation.

* *P* < .05 versus NGT by logistic regression analysis.

** *P* < .01 versus NGT by logistic regression analysis.

*** *P* < .0001 versus NGT by logistic regression analysis.

## 4. Discussion

The present study examines impacts of IPH on 10-year ASCVD risk scores estimated by using the Pooled Cohort Equations in women after menopause, comparing the differences in predicted ASCVD risk while the hidden DM is detected or concealed. Our data demonstrated that identification of hidden DM not only resulted in a significant increase (almost 2-fold) in predicted risk for the IPH women (Fig. 1, scenario 2) but also led to a shift to a higher 10-year ASCVD risk category in 10% of the study subjects (45 out of the 428 subjects) (Table 2 and Fig. 2). Half of the reclassified subjects were reallocated from a lower risk category to a category of 10-year risk ≥7.5%. The AHA/ACC guidelines recommend high-intensity statin therapy for DM patients (40–75 years of age and LDL cholesterol 70–189 mg/dL) with a 10-year risk of ASCVD events ≥7.5%.^[2]^ Moderate-intensity statin therapy is recommended for DM patients with lower risk.^[2]^ For those who were re-categorized from low to the category of 10-year risk ≥7.5%, the reclassification altered patient eligibility for cholesterol-lowering agents. The unique clinical circumstances of IPH may expose the complexity of using Pooled Cohort Equations to discuss risk-benefit of statin therapy with patients in everyday practice.

There have been concerns about the robustness of risk estimates using the Pooled Cohort Equations. Risk equations are mathematical models developed from results of prospective cohorts to estimate risk of outcomes in an individual. Inherent limitations always exist when applying group equation to a single person.^[13]^ Estimates of risk are conditioned on the information included in the risk calculation. When the risk of each subject was recalculated based on additional information, extensive reclassification may occur accordingly.^[13]^ Patient demographic characteristics remain an important point as well when considering the usefulness of risk calculators.^[4]^ For example, many risk calculators do not consider central obesity as a risk component. Ancheta et al^[14]^ demonstrated that increased waist circumference was associated with increased cardiovascular risk scores in Filipino-American women. In that particular group of women, the contribution of central obesity to estimated cardiovascular risk may be underestimated.

Additionally, Gupta et al^[5]^ employed the National Health and Nutrition Examination Survey dataset (2005–2010) to compute maximum and minimum 10-year risks by assuming variations occurred in related risk components. They found that uncertainties in input of age (0–1 years) and ±10% variation in BP, total cholesterol, and HDL cholesterol values for calculating 10-year risk scores would alter risk categorization in up to 24% of the study subjects. Here, we describe the input uncertainty of DM status (0 or 1) on 10-year ASCVD risk scores in postmenopausal women with IPH. Approximately 20% women with IPH were re-categorized from risk category of <5% to ≥7.5% once the women were identified as DM (Fig. 2). The current analysis along with results from Gupta et al provides a critical caveat that needs to be concerned in risk calculation for individuals at risk of DM, which is, input uncertainties may have significant impacts on the calculated 10-year risk.

Since 2015, the lipid management recommendations for type 2 DM from the American Diabetes Association (ADA) are concordant with the AHA/ACC guidelines.^[15]^ However, the current ADA’s Standards of Care do not consider using Pooled Cohort Equations for assessing cardiovascular risk in individuals with DM.^[16]^ Instead, the ADA guidelines stratify DM patients by age (<40, 40–75, and >75 years) and ASCVD risk factors (including LDL cholesterol >100 mg/dL, high BP, smoking, chronic kidney disease, albuminuria, and family history of premature ASCVD) for choosing statin therapy for primary prevention. Recently, Bouchonville et al^[17]^ critically reviewed the evidence behind the ADA guidelines on the prevention of cardiovascular disease in DM. The authors argued that the evidence to support recommendations of the ADA guidelines regarding statin therapy in DM was incomplete. Although the ACC/AHA and the ADA guidelines acknowledge that not all patients with type 2 DM have equivalent risk, how to initiating statin therapy based on risk estimation is still a major challenge to treating physicians.

People with IPH warrant study because these people had the same risk of cardiovascular death^[18]^ and total mortality^[19]^ as those who were known to have DM. Liu et al^[20]^ suggested that high cardiovascular risk in individuals with DM was largely driven by coexisted multiple cardiometabolic derangements. In the present study, we found that postmenopausal women with IPH were already characterized by unfavorable cardiovascular risk profile and high 10-years ASCVD risk scores, regardless of whether the hidden DM was detected (Table 1 and Fig. 1). Considering approaches to primary prevention for ASCVD, results of our study emphasize the need to focus on clustering of metabolic abnormalities accompanying IPH, not just on postchallenge hyperglycemia per se. Consistent with other studies,^[10,18,19]^ women with IPH in the present study were older and had higher BP, higher TG and lower HDL cholesterol values than women without IPH (Table 1). The finding that women with IPH had a higher 10-year risk score than their counterparts (Fig. 1, scenario 1) might be a combined effect of aging, high systolic BP, and low HDL cholesterol on ASCVD risk. Notably, the constellation of low HDL cholesterol and high BP in elderly is not a benign condition. Kim et al^[21]^ reported that presence of low HDL cholesterol with high-normal BP in subjects older than 60 years would independently increase total mortality by about 2-fold. The subset of postmenopausal women with IPH who also have high BP and low HDL cholesterol may be exceptionally at increased risk of fatal disease. This hypothesis could be a topic of future investigation.

Numerous studies have shown associations between postmenopausal status and elevated levels of total cholesterol and LDL cholesterol.^[22,23]^ Although levels of total cholesterol and LDL cholesterol were similar among the 3 groups in this study (Table 1), the mean LDL cholesterol levels were higher than 130 mg/dL in all groups studied (Table 1) and were above recommendations.^[16,24]^ Our data reflect the fact that the 3 study groups were all under the influence of postmenopausal status. The results of the present study by no means indicate that total cholesterol and LDL cholesterol are not important risk factors for ASCVD for postmenopausal women with IPH. Total cholesterol just appeared contributing equally to women with or without IPH while calculating 10-year risk scores in the current study.

The present study is limited to postmenopausal women only. Our results may not be generalized to younger women or to men. DM are highly prevalent in midlife women and about half of those are unaware of their diagnosis.^[8,24]^ Physicians are supposed to be aware of the uncertainty described in the present study. Moreover, some remaining limitations of the Pooled Cohort Equations need to be considered.^[3,4]^ The Pooled Cohort Equations apply most accurately to non-Hispanic Whites and African Americans. For non-White and non-African American ethnic groups, the equations for Whites of the same sex were used, which may lead to unpredictable over- and underestimation in other ethnicity groups.^[3]^ Yang et al^[25]^ found that the Pooled Cohort Equations overestimated risk for Chinese men and underestimated risk for Chinese women. The absence of other ethnicities in the data sources limits the applicability of the Pooled Cohort Equations to other populations, such as Chinese.^[3]^ Third, prediction of ASCVD risk in people with IPH is poorly understood. The current study is a cross-sectional study. It is not our intention to validate the Pooled Cohort Equations for postmenopausal women with IPH. Considering type 2 DM does not produce specific symptoms for many years and the clustering of vascular risk associated with DM appears early during the development of type 2 DM,^[24]^ we discuss the ambiguity created by the current binary input of DM status in the Pooled Cohort Equations. Gupta et al^[5]^ proposed that a new version of the risk calculator should estimate upper and lower boundaries for 10-year ASCVD risk taking into account various uncertainties in input variables.

## 5. Conclusion

In conclusion, the 2013 ACC/AHA guidelines identified persons who will benefit from statin therapy for primary prevention by age, with or without DM, and 10-year ASCVD risk higher or lower than 7.5%. The present study showed that identification of hidden DM in postmenopausal women with IPH would significantly increase their predicted ASCVD risk calculated by the Pooled Cohort Equations. Furthermore, the uncertainties in the dichotomous input variable of DM status (yes or no) could alter patient eligibility for cholesterol-lowering agents for women with IPH.

## Author contributions

**Conceptualization:** Yi-Chun Lin, Chii-Min Hwu.

**Data curation:** Tsung-Hui Wu, Yi-Chun Lin, Chii-Min Hwu.

**Formal analysis:** Tsung-Hui Wu, Yi-Chun Lin, Chii-Min Hwu.

**Investigation:** Tsung-Hui Wu.

**Methodology:** Tsung-Hui Wu, Chii-Min Hwu.

**Supervision:** Chii-Min Hwu.

**Validation:** Yi-Chun Lin, Chii-Min Hwu.

**Writing – original draft:** Tsung-Hui Wu, Yi-Chun Lin, Chii-Min Hwu.

**Writing – review & editing:** Chii-Min Hwu.
